# Learning how to explore spiritual aspects in encounters with patients with chronic pain: a pre-test post-test trial on the effectiveness of a web-based learning intervention

**DOI:** 10.1186/s12909-024-06142-2

**Published:** 2024-10-25

**Authors:** Felix Michael Schmitz, Ann-Lea Buzzi, Beate Gabriele Brem, Kai Philipp Schnabel, Joana Berger-Estilita, Fredy-Michel Roten, Simon Peng-Keller, Sissel Guttormsen

**Affiliations:** 1 Institute for Medical Education, University of Bern, Mittelstrasse 43, Bern, 3012 Switzerland; 2 Department of Anesthesiology and Pain Medicine, Bern University Hospital, University of Bern, Bern, Switzerland; 3 Valais Cantonal Rescue Organization: Sierre, Valais, Switzerland; 4 Spiritual Care, University of Zurich, Zurich, Switzerland

**Keywords:** Spiritual resources, Spiritual distress, Communication skills, Education, Training, Spiritual assessment, Chronic pain, Patient simulation, Pre-test post-test trial

## Abstract

**Background:**

Integrating spiritual aspects into treatment plans can release essential resources for coping with chronic pain. However, some spiritual aspects may also induce distress and hinder the coping process. There is a lack of evidence regarding how to perform the clinical tasks of exploring spiritual aspects and of instruments assessing related competencies. Therefore, we developed a web-based learning module to provide education on the subject alongside corresponding assessment instruments. The module presents the InSpiRe (Integration of Spirituality and/or Religion in patient encounters) protocol. The instruments encompass cognitive, affective, and behavioral dimensions.

**Methods:**

This paper aims to determine (i) the learning effects associated with completing the web-based spiritual-care learning module and (ii) the reliability and validity of the instruments employed. To address these aims, we conducted a pre-test/post-test trial with *N* = 32 randomly selected fourth-year medical students. During the pre-test, we assessed the students’ knowledge, attitudes, and self-efficacy regarding exploring spiritual aspects. For this purpose, we developed a short-answer knowledge test, an attitude questionnaire, and a self-efficacy scale. Additionally, the students explored spiritual aspects with a simulated patient portraying a person with chronic pain. Three trained raters evaluated the students’ performances using a self-developed scale. In the intervention phase, the students completed the 45-minute learning module on a personal computer. The module presented InSpiRe-related content as text and step-by-step video demonstrations, including hints that denote critical actions. The subsequent post-test was identical to the pre-test.

**Results:**

The internal consistency was suitable for all respective instruments, and there was an indication of solid validity of the performance test. After completing the spiritual care learning module, the students showed statistically significant increases in knowledge scores and significant positive shifts in their attitudes and levels of self-efficacy regarding exploring spiritual aspects. They also attained significantly higher performance scores in the same regard.

**Conclusions:**

Completing the spiritual-care module is associated with meaningful learning effects on cognitive, affective, and behavioral dimensions related to exploring spiritual aspects, as demonstrated in the post-test conducted shortly after the intervention. Due to good reliability and validity scores, the self-developed instruments can be applied appropriately.

**Supplementary Information:**

The online version contains supplementary material available at 10.1186/s12909-024-06142-2.

## Background

 There is a growing consensus among researchers, politicians, clinicians, and patients that spiritual aspects should be considered in various healthcare services [1–4]. Spiritual care is particularly relevant for patients with chronic pain. If medication is no longer effective, health professionals should support the search for other strategies for coping with the pain [5]. Spiritual aspects, which broadly address inherent questions regarding one’s meaning and purpose in life [6], are known to impact the experience and processing of chronic pain for many patients [7].

The effects of some spiritual aspects on the coping process are known to be positive, while other elements can impede functional coping. Evidence shows that the belief that there is ultimate meaning in life and a sense of spiritual connection with others [8] leads to higher self-esteem, a better quality of life and psychological adjustment [9, 10]. Access to such positive aspects – hereafter referred to as *spiritual resources* – is associated with functional pain-related coping, leading to, amongst other things, improved pain tolerance [11]. On the other hand, viewing pain or disease as punishment [12] is related to depression, emotional distress, poor quality of life, and poor problem resolution [8, 13]. Such negative aspects – hereafter referred to as *spiritual distress* – can worsen the perception and processing of chronic pain [9, 14]. Moreover, suffering from pain may further increase one’s spiritual distress [15, 16].

Although various spiritual aspects critically impact general well-being and pain-related coping processes, spirituality is usually perceived as personal and private. This is one reason why many health professionals do not regard addressing the patients’ potential spiritual resources and distress as part of their duties [17]. Another reason is that professionals are uncertain about how to perform this task [18]; they perceive a lack of communication skills to address spiritual aspects adequately and, therefore, avoid raising them [19]. Hence, there is a need for sensitization to the possible (positive or adverse) influence of spiritual aspects on health-related outcomes, which can be achieved by providing a profound knowledge base on the issue and practical guidance on how to explore spiritual aspects proficiently during patient encounters [20].

We acknowledge the existence of well-established instruments for (self-)assessing spiritual care competencies, such as the Spiritual Care Competence Scale (SCCS) [21] for nurses, which offer valuable insights into general spiritual care proficiency. However, assessing specific competencies related to exploring spiritual resources and distress during patient encounters is not fully covered by existing scales. Thus, instruments that assess these task-related competencies are also required.

To address those needs, we developed a web-based learning module [22] providing critical information, including descriptions of spirituality and spiritual care and the potential effects on pain-related coping processes. The module presents a protocol covering how spiritual resources and spiritual distress can be explored in patient encounters, i.e., the InSpiRe (*Integration of Spirituality and/or Religion in patient encounters*) protocol. The nine steps covered in InSpiRe were compiled from the literature, adapted for German-speaking countries, and revised in a focus-group study with experts in the field [23]. In addition, due to the lack of existing scales, we developed accompanying instruments to measure InSpiRe-related competencies.

### Key concepts

To ensure clarity and comparability in our study, we define several key concepts used throughout the research.

#### Coping

In this context, coping refers to the cognitive and behavioral efforts made by individuals to manage the stress, emotions, and challenges posed by chronic pain. Coping strategies may involve problem-solving, emotional regulation, or seeking social and spiritual support. Also, religious coping, as defined by Pargament et al. [8], which consists of the use of religious beliefs and practices to manage stress, can influence both emotional well-being and physical outcomes in the context of chronic pain. Positive religious coping strategies include engaging in prayer, while negative coping may involve feelings of abandonment by a higher power.

#### Spirituality

For this study, we define spirituality broadly as the search for meaning, purpose, and connection, which may or may not include a religious dimension. Spirituality can encompass beliefs, practices, or experiences that contribute to an individual’s sense of well-being and ability to cope with life’s challenges, particularly in the context of chronic illness.

#### Spiritual aspects

When we refer to ‘spiritual aspects’, we mean specific elements of a person’s spirituality, including their beliefs, practices, and values related to, e.g., meaning, purpose, and connection. For instance, meaning in life is understood as an individual’s sense of purpose or understanding of their existence, particularly in response to chronic illness or pain. Patients who find meaning in their experiences often demonstrate greater resilience and an enhanced ability to cope with the challenges of chronic pain. Connection with others is a spiritual concept that refers to the sense of belonging and support individuals experience through relationships or community involvement. For patients with chronic pain, this connection can provide essential emotional and spiritual support, fostering a greater ability to manage their condition. While these aspects are integral to an individual’s spirituality, they are discussed as distinct components that healthcare providers can explore during clinical encounters. In this way, ‘spiritual aspects’ offer a practical framework for addressing spirituality within a medical context, as they can positively or negatively influence coping with pain.

#### Spiritual care

Spiritual care involves the support provided by healthcare professionals to address the spiritual needs of patients, recognising the potential influence of spirituality on health outcomes. It includes exploring spiritual resources (e.g., faith, hope, meditation) and spiritual distress (e.g., loss of meaning or feeling punished) during patient encounters.

#### Chronic pain

We define chronic pain as persistent pain lasting longer than three months that negatively impacts the patient’s physical and emotional health and quality of life. Chronic pain is often multifactorial and requires a holistic treatment approach, including attention to the psychological and spiritual dimensions of patient care.

#### Web-based learning module

In this study, the web-based learning intervention refers to the online educational module (InSpiRe) developed to teach medical students how to conduct spiritual assessments in patient encounters. This module includes text-based instruction, video demonstrations, and interactive elements designed to enhance the learning of spiritual care concepts. (The module is further described in the Learning intervention: completing the web-based learning module section).

## Methods

With this study, we aimed to examine how the web-based spiritual care learning module impacts learners. To clarify, we investigated cognitive, affective, and behavioral dimensions involved in exploring spiritual aspects in encounters with patients with chronic pain using new, self-developed instruments.

The following hypotheses were tested.

- *Hypothesis 1: After completing the learning module, learners will achieve higher knowledge scores in exploring spiritual aspects in patient encounters.*
- *Hypothesis 2a: After completing the module, learners will positively shift their attitudes towards exploring spiritual aspects.*
- *Hypothesis 2b: After completing the module, learners will perceive higher levels of self-efficacy in exploring spiritual aspects.*
- *Hypothesis 3: After completing the module, learners will attain higher performance scores in exploring spiritual aspects during a simulated encounter.*

In addition, we aimed to determine the reliability and validity of the instruments used, where applicable, to ensure that the findings are reasonable.

**Fig. 1:**
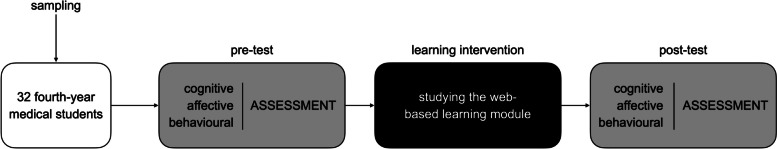
Flow of participants

To address our aims and to test our hypotheses, we performed a pre-test/post-test trial with randomly selected participants; data were recorded before and after participants were tasked with completing the web-based learning module (learning intervention). Figure 1 illustrates the study flowchart. Ethics approval was obtained from the independent cantonal ethics committee before data collection (for details, see Ethics approval and consent to participate).

The study took place at the skills lab of the Medical Faculty at the University of Bern. So, the study was conducted within the Swiss medical education system, where cultural and religious diversity is prevalent. (The learning module was designed with adaptability, making it applicable across various cultural contexts). We equipped one test room with two video cameras and a microphone (for recording the simulated encounters, see Behavioral assessment: performance test section), a computer with an internet connection, a mouse and keyboard, and a widescreen monitor (for completing further tests and the module, see Pre- and post-tests section). Participants were tested individually. Each participant spent approximately two hours in the experiment.

### Sampling

The sampling method for our study was designed to ensure randomness and mitigate selection bias. A power analysis using G*Power (https://www.psychologie.hhu.de/arbeitsgruppen/allgemeine-psychologie-und-arbeitspsychologie/gpower) was conducted based on the anticipated effect sizes for changes in knowledge, attitudes, and behaviors following the educational intervention. Given the within-subject design, a sample size of *N* ≥ 30 participants was considered sufficient to achieve large effects at a significance level of 0.05, while also adhering to the Central Limit Theorem.

Thirty-six out of 102 fourth-year medical students were randomly selected and assigned to attend a mandatory internship at the Department of Anesthesiology and Pain Medicine at Bern University Hospital using the RANDOM INTEGER GENERATOR (https://www.random.org/integers/). Of these, *N* = 32 students (18 females and 14 males, aged between 22 and 33 years (*M* = 24.2, *SD* = 2.2)) agreed to participate, resulting in a participation rate of 88.9%. All participants provided written informed consent for the use of collected data for research purposes and confirmed that they had not been exposed to the learning materials before participation. (While reasons for non-participation were not detailed, the high opt-in rate suggests strong engagement with the study).

### Pre- and post-tests

Pre- and post-tests comprised the same assessments ensuring comparability between the pre- and post-intervention data. Each test lasted approximately 30 minutes: 15 minutes for the cognitive and affective evaluations and 15 additional minutes for the behavioral assessment. The post test was conducted immediately after the completion of the web-based learning module. This timing was chosen for pragmatic reasons, as it allowed us to measure the immediate learning effects of the intervention without introducing additional variability due to scheduling conflicts or time delays. The assessments are described in the following sections.^1^

#### Cognitive assessment: knowledge test

Participants described their knowledge of spiritual care by answering a questionnaire with six open-ended questions (Appendix A) before and after the learning intervention. We collected participants’ replies in the UNIPARK survey tool (http://www.unipark.info) running on the computer in the test room.

After data collection, two experts—medical doctors with years of experience treating patients with chronic pain and holding a Swiss CAS in spiritual care—scored every answer. They were familiar with the taught protocol (see InSpiRe protocol: learning materials provided section) and received standardized training on how to rate the answers by assigning points on a scale from 0 to 3, where *0 = no reply/wrong answer* and *3 = excellent*, ensuring the most accurate outcomes. The raters scored the answers independently and in a randomized order. They were blinded to the point of measurement (pre-test or post-test) and to any data related to the authoring participants (pseudonymized answers). Inter-rater reliability was assessed to ensure consistency in their evaluations.

We based each participant’s final pre- and post- knowledge score, which could vary between 0 and 18 points, on the average scores derived from the two experts’ assessments.

#### Affective assessment: attitude and self-efficacy

**Fig. 2:**
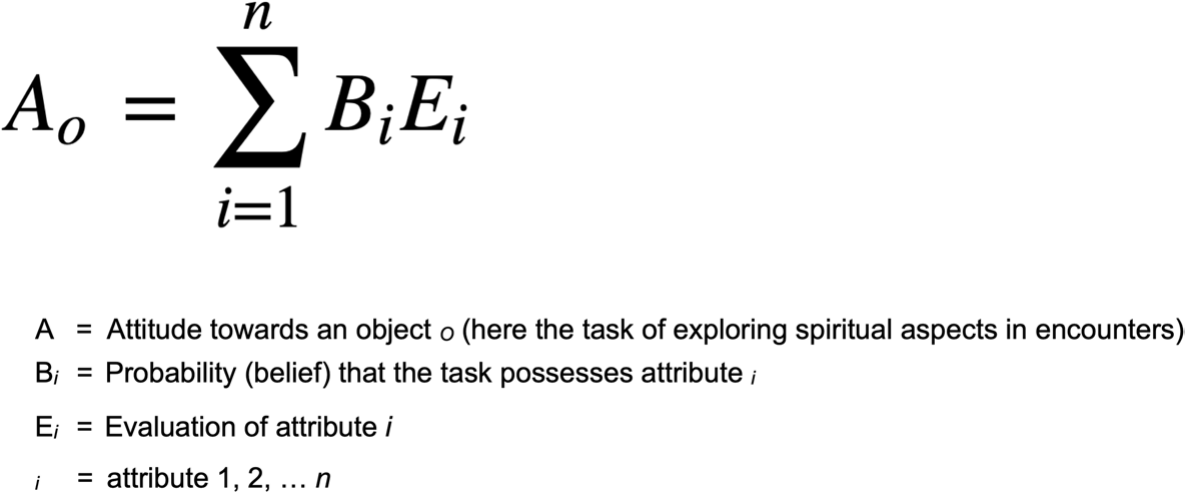
Fishbein and Ajzen’s formula to calculate the overall attitude towards a given object

We recorded pre- and post-interventional participants’ attitudes towards exploring spiritual aspects in patient encounters using an eight-item paired questionnaire (Appendix B). Following the approach from Fishbein and Ajzen [24], each item pair comprised one statement covering the probability of occurrence of a particular attribute that might be related to the issue (e.g., willingness to talk about spiritual aspects) and one statement addressing the connotation of the same attribute (e.g., perceived importance of willingness to talk). The participants’ overall attitude scores were calculated according to the Fishbein and Ajzen formula (Fig. 2). Final scores ranged from − 64 to + 64 points, with higher scores indicating a more favorable overall attitude towards exploring spiritual aspects.

Based on Bandura’s work [25], we used a one-item scale to indicate the participants’ self-efficacy for performing the task independently. The item used a five-point Likert scale to adequately cover the extent to which participants had trust in their ability to explore spiritual aspects in a subsequent simulated encounter. Both affective assessments were also input into the UNIPARK survey tool.

#### Behavioral assessment: performance test

Finally, we requested participants to take a problem-oriented medical history with a simulated patient (SP) portraying a person with chronic pain. With this test, we aimed to assess how successfully the participants accomplished exploring spiritual aspects, particularly in identifying potential spiritual resources and spiritual distress of the SP. We used two chronic pain-related scenarios (Appendices C and D), in which the SP was trained to reply like “*Sometimes I wonder if it all makes sense*,* especially when I wake up in the morning with such severe pain*”, “*I suffer from the inability to achieve my meditative states through physical activity*”, and “*I can find some inner peace in nature*,* and drawing sometimes helps me forget the pain for a moment*”.^2^ A multidisciplinary team with expertise in chronic pain management and medical education developed the scenarios.

The scenarios were performed in a counterbalanced order to avoid rank order effects, with half of the participants performing one scenario in the pre-test and the other in the post-test. The opposite order was applied to the other half of the participants. The SP encounters took place in the same test room but at a separate table with two chairs arranged in the two preinstalled cameras’ focal points between the two speakers.

All SP encounters were video recorded. Three outcome raters (Masters-level psychology students) independently rated every participant’s skills performance, including the performances shown in the post-test. Following the procedure from Schmitz et al. [26], the raters used an assessment scale developed for this purpose (InSpiRe Assessment Scale; Appendix E) and were given adequate training in its use. Raters were blinded to the point of measurement (pre- vs. post-test), the learning intervention, and our hypotheses until they had completed the entire scoring. The participants’ final performance scores were calculated as grand means based on the three raters’ evaluations. Scores between 1 and 5 points could be achieved.

Since exploring spiritual aspects is regarded as a task that is anchored in the domain of patient communication [19] and since there is a lack of published instruments addressing this particular task, the three outcome assessors additionally rated the participants’ general communication skills by using the validated Berlin Global Rating scale (BGR) [27]. The BGR scale is a global rating tool for assessing communication skills in medical encounters. It includes items such as organization of the encounter, interpersonal skills, and overall communication effectiveness. Each item is rated on a Likert scale, and the final score reflects the assessors’ overall impression of the participant’s communication competence. This approach allowed for drawing conclusions about the validity of the self-developed InSPiRe Assessment Scale (see Statistical analysis and Cognitive assessment outcomes sections).

### Learning intervention: completing the web-based learning module

**Fig. 3:**
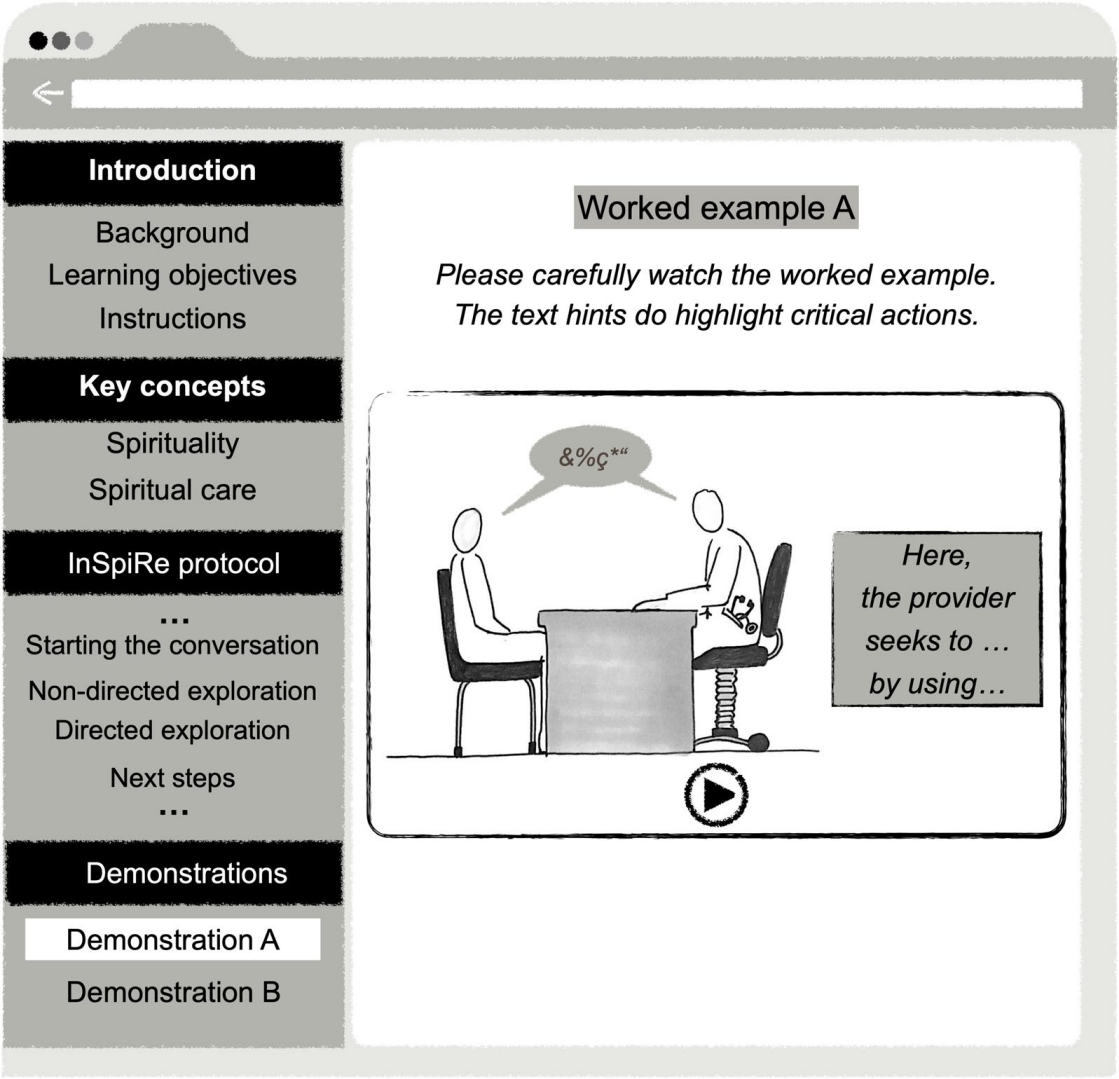
Simplified representation of the web-based spiritual care learning module. *Note*: Besides the text instructions, two 10-minute audio-video demonstrations were implemented, one showing a patient with chronic daily headaches and one with acute pain due to a hernia (and the surgical intervention being delayed). Both patients, played by actors, talked to a provider seeking to support them in functionally coping with the pain by exploring spiritual resources and distress as provided by the InSpiRe protocol. Each demonstration was accompanied by 16 text hints denoting the critical actions of the provider according to the corresponding steps of InSpiRe. The hints comprised between 13 and 91 words

Between the pre-and post-tests, we gave participants 45 minutes to complete the web-based spiritual care learning module. Figure 3 shows the module’s didactic design. This module addressed the primary learning objective of introducing the InSpiRe protocol and laying the ground for the practical use of its steps in respective encounters.

#### InSpiRe protocol: learning materials provided

All nine steps of the InSpiRe protocol were explained as running text in the module (see Table 1). As four of the steps are to be performed during actual encounters, they were additionally exemplified with audio-video demonstrations, built as worked examples following Clark, Nguyen, and Sweller [28]^3^. According to Lorch [29], the examples were accompanied by text hints that denoted critical actions related to the steps of the InSpire protocol (see Fig. 3). Evidence has shown that educational tools scaffolded in this manner can foster the initial learning of clinical communication skills, such as breaking bad news [26, 30]. This approach reduces cognitive load and promotes the acquisition of cognitive schemas, enabling providers to readily act upon the requested communication principles (cf. [28]).

**Table 1:** Overview of the InSpiRe steps, their underlying actions, and principles

Stage	Step	Actions	Principles (examples^a^)
***Before the encounter***	***Indication***	Reflecting on whether spiritual aspects could be important for patients with chronic pain.	- Pain can trigger questions of sense and meaning. - Pain can be experienced as punishment. - These aspects can influence how individuals cope with pain and should therefore be addressed.
	***Preparation***	Planning related to time, location, and participants.	- Considering taking time for the exploration of potential spiritual aspects during the consultation with the patient. - Arranging for some privacy can put the participant at ease and support the patient’s willingness to talk about his/her spirituality. - Providing the opportunity to discuss the topic in the presence of a trusted friend or relative can deliver additional support.
	***Self-reflection***	Reflecting on the potential impact of a patient’s possible spiritual resources and/or distress on one’s own personal beliefs.	Being aware of one’s own beliefs and related trigger points can help health care providers to open up for the patient’s faith and to accept if his/her faith is fundamentally different from his/her own faith or beliefs.
***During the encounter***	***Starting the conversation***	Welcoming the patient; opening the conversation in an open, indirect, or direct way; seeking for consent.	*“The last time we talked*,* you mentioned*,* that you wonder why God punished you in this way. Your statement made me think*,* and I wonder if it would be okay for you to talk with me about that in more detail?”*
	***Non-directed exploration***	Exploring spiritual aspects by using open-ended questions about (non-)functional coping; listening; mirroring	*“I wonder*,* if there are aspects [in spirituality] that give you strength.”*
	***Directed exploration***	Identifying spiritual resources/distress by asking specific spirituality-related questions.	*“So*,* if I hear what you are telling me*,* the weekly walk through the forest could be an important source of strength for you – is that correct?*
	***Next steps***	Determine future steps aligned with spiritual aspects that could enhance/worsen the patient’s pain-related coping: (A) no more action necessary, (B) follow-up conversations, (C) referral to specialist (e.g., chaplain or psychotherapist), (D) specific measures (e.g., mindfulness-training or church visiting programs); summarizing the main points; ending the conversation using acknowledging words and ensuring availability for potential future encounters.	*“As we discussed*,* it seems like you would be open to try a training in mindfulness as an additional option in your treatment plan. Maybe*,* you give it a try and we meet again after your first sessions to see if this works out for you.”*
***After the encounter***	***Review***	Reflecting whether the most important aspects have been addressed; checking the own emotional state.	- *How did the conversation go? Were all aspects that seem significant addressed?* - *Am I satisfied with the course of the conversation? What went well? What could I do differently and perhaps better next time?* - *How did I feel at the end? Were there moments in the conversation when I felt uncomfortable? Which of my own issues were triggered in the conversation with the patient?*
	***Organization***	Organizing follow-up visits, referrals, treatments, …	There might be nothing further to organize after a given encounter. However, it is worthwhile to pause for a moment and consider which measures have been reflected on with the patient.

^a^Some actions are exemplified instead of delivering principles in the rightmost column, which is especially true for the stage “during the encounter”

### Statistical analysis

The statistical analysis used the Statistical Package for Social Sciences (SPSS) version 28. To determine the reliability of our instruments, we relied on measures indicating their internal consistency and, if applicable, the inter-rater agreement. Specifically, we calculated Cronbach’s Alphas (*α*) for the knowledge, attitude questionnaire^4^, and performance tests. Following Steiner [31] *α*s above 0.7 and below 0.9 are most suited for empirical studies (in his opinion, *α*s over 0.9 would most likely indicate unnecessary redundancy rather than a desirable level of consistency). In addition, we calculated intraclass correlation coefficients (*ICC*) [32] for the knowledge and the performance test. According to Koo et al. [33], *ICC* measures below 0.5 imply low, from 0.5 moderate, 0.75 good, and 0.9 excellent agreements. To infer the validity of the performance test, we calculated Pearson correlations (*r*) between the final scores based on the InSPiRe-Assessement-Scale and on the BGR. According to Cohen [34], coefficients between |*r*|=0.1 and |*r*|=0.3 imply weak, between |*r*|=0.3 and |*r*|=0.5 mediocre, and |*r*|>0.5 strong correlations.

To test our hypotheses, we calculated paired samples *t*-tests with statistical significance determined at the 5% level. Effect sizes were determined using Cohen’s d (*d*), implying minor effects from 0.2, medium effects from 0.5, and large effects from 0.8 [34].

## Results

### Reliability and validity

The internal consistency was suitable for all respective instruments, i.e., the knowledge test (*α* = 0.85), the attitude questionnaire (*α* = 0.88), and the performance test (*α* = 0.72). Inter-rater reliability was excellent for the knowledge test (*ICC* = 0.94) and good for the performance test (*ICC* = 0.89). Ultimately, there was an indication for solid (convergent) validity of the performance test (InSPiRe) as the correlations between its scores and those from the BGR scale were strong (Table 2). We conclude that our self-developed instruments can be applied appropriately.

**Table 2:** Pearson correlation coefficients between the InSpiRe-Assessment-Scale and the BGR^a^ scores

	Pre	Post	Averaged
*r*	0.70**	0.62**	0.76**
*n*	32	32	32

Note: **p < 0.01, two-tailed

^a^The inter-rater agreement for the BGR scale was good (*ICC* = 0.88)

### Cognitive assessment outcomes

**Fig. 4:**
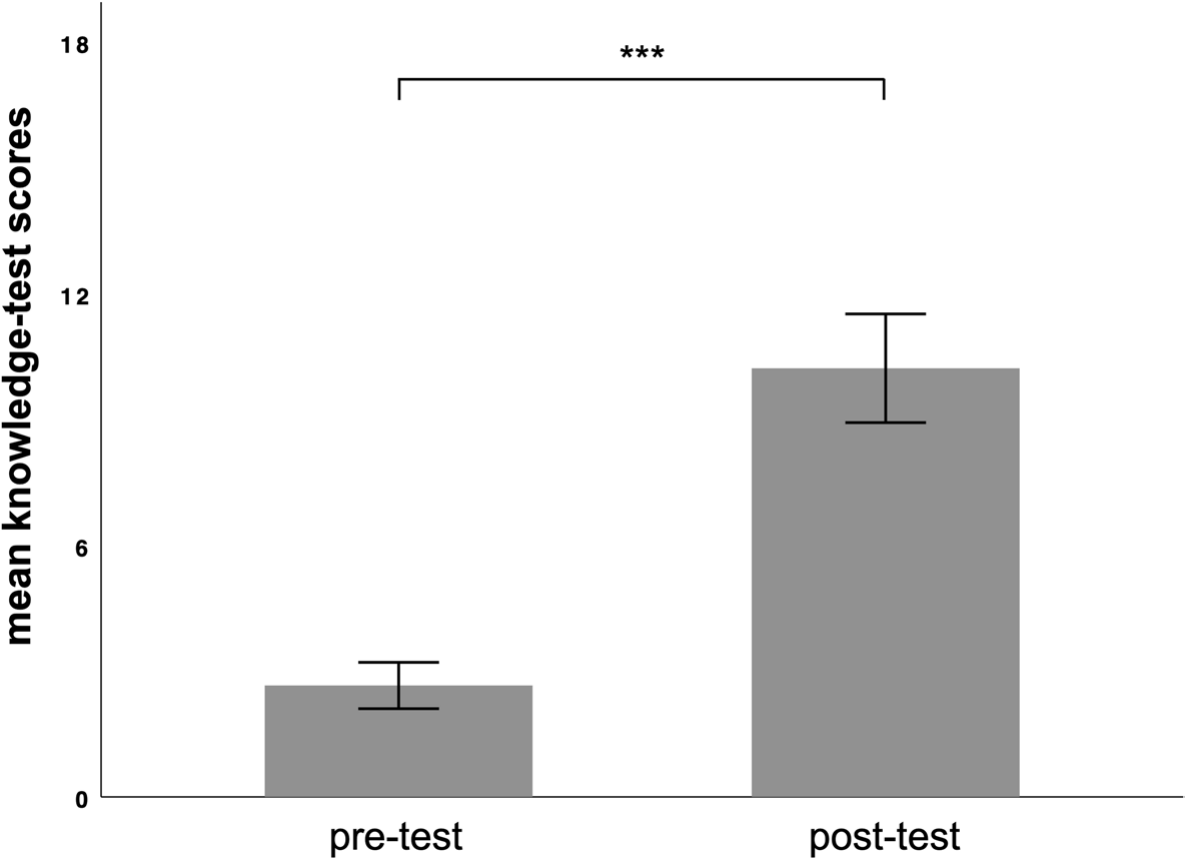
Bar graph showing the mean knowledge scores from the pre-test and the post-test. The error bars represent the 95% confidence interval. *Note*: ***p < 0.001, two-tailed

The knowledge test results are shown in Fig. 4. Our analysis revealed that, compared to the pre-test, participants attained significantly higher knowledge-test scores after undergoing the learning intervention *(|t|(df = 31)* = 12.2, *p* < 0.001; *d* > 1). Hypothesis 1 was supported.

### Affective assessment outcomes

**Fig. 5:**
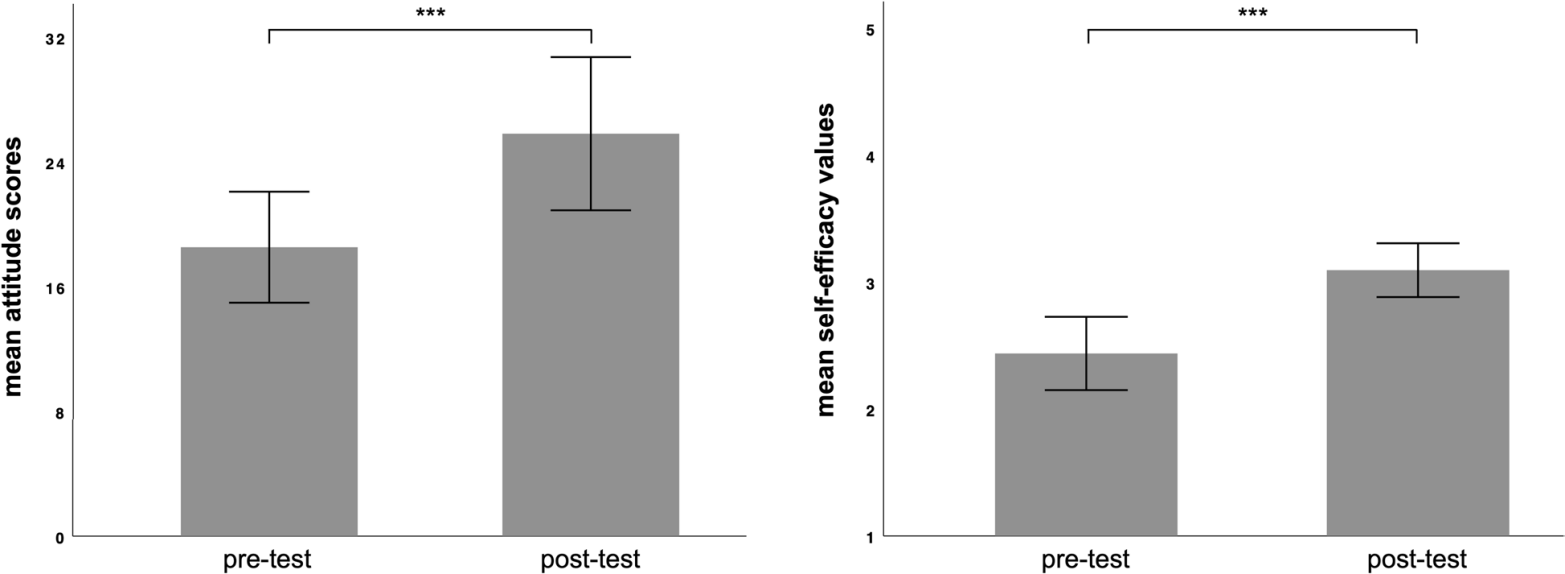
Bar graphs showing the mean attitude scores (left) and the mean self-efficacy values (right) derived from the pre-test and post-test. The error bars represent the 95% confidence interval. *Note*: ***p < 0.001, two-tailed

Figure 5 illustrates the results of the two affective assessments derived from the pre- and the post-tests. Participants demonstrated a significantly positive shift in their overall attitude towards exploring spiritual aspects after completing the web-based learning module (|*t*|(*df* = 31) = 5.47, *p <* 0.001; *d* = 0.97). The analysis further showed that the participants’ self-efficacy for performing this task increased significantly after the learning intervention (|*t*|(*df* = 31) = 4.72, *p <* 0.001; *d* = 0.80). Hypotheses 2a and 2b were supported.

### Performance assessment outcomes

**Fig. 6:**
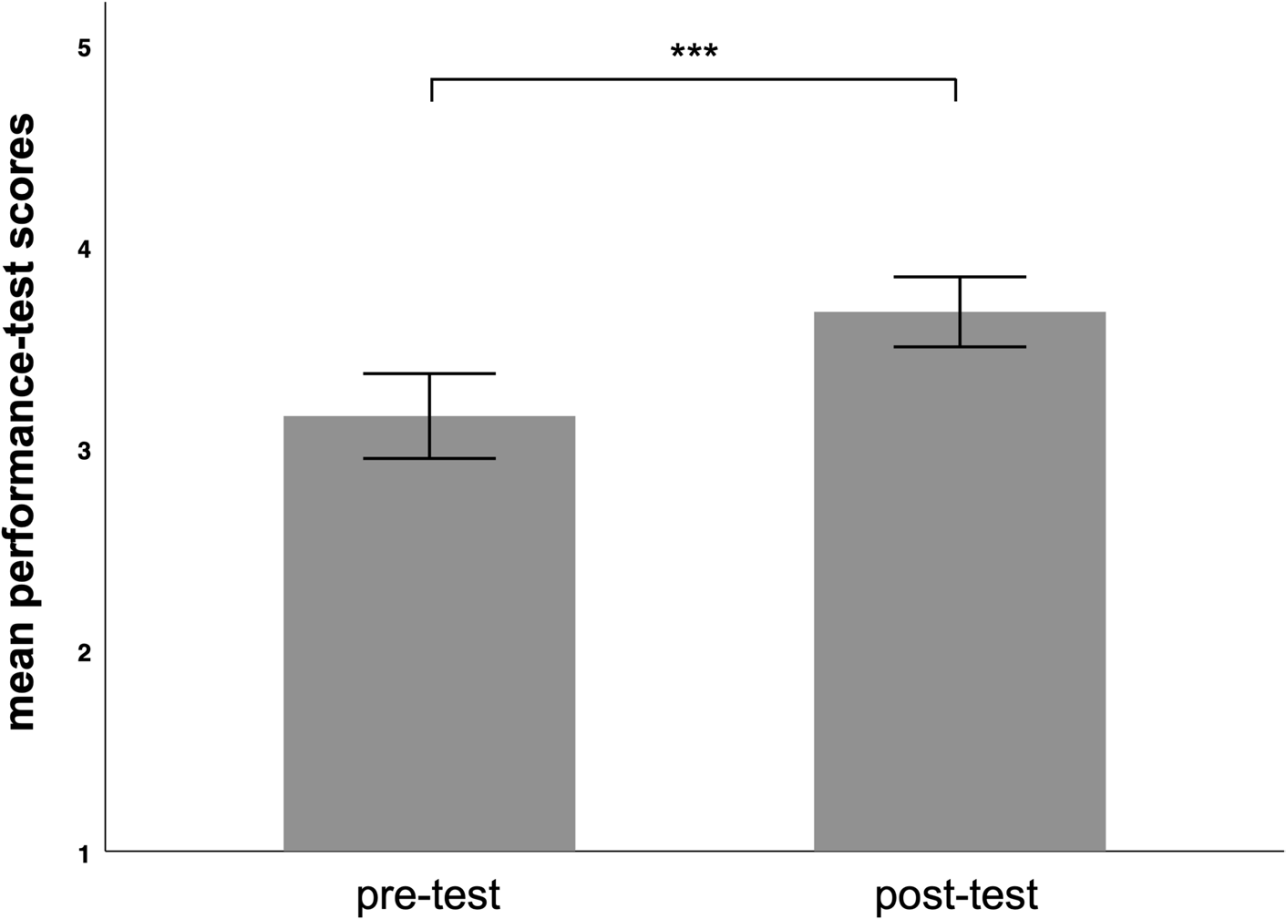
Bar graph showing the mean performance-test scores from the pre-test and the post-test. The error bars represent the 95% confidence interval. *Note*: ***p < 0.001, two-tailed

Participants achieved mediocre performance-test results in the pre-test and improved results in the post-test (Fig. 6). Analysis showed that this increase was significant ((|*t*|(*df* = 31) = 4.69, *p <* 0.001; *d* = 0.82). Hypothesis 3 was supported.

## Discussion

Chronic pain is acknowledged as a multifactorial experience negatively impacting the patients’ psychosocial health and quality of life. Therefore, a multimodal treatment approach to pain should also address spiritual aspects, i.e., spiritual resources (e.g., practices like meditation or beliefs and expectations like hope) and spiritual distress (e.g., loss of hope or feeling punished). However, addressing spiritual resources and distress in patient encounters remains rare and mostly non-standardized [35]. Völz et al. [36] noted that healthcare professionals often express uncertainty about their roles and responsibilities when addressing spiritual aspects, with many feeling unprepared or hesitant to engage in such conversations. Our study underscores the importance of equipping providers with the skills and confidence to address spiritual resources and distress, aligning with the broader effort to integrate spiritual care into medical practice. The spiritual care learning module was designed to bridge this gap by offering practical tools and education that empower medical students to engage with the spiritual aspects of patient care confidently. Against these backgrounds, the presented pre-post study on the effectiveness of the web-based learning intervention delivers evidence that it is possible to sensitize healthcare professionals to this critical communication task and that its steps can be learned and applied successfully to practice.

Our study revealed the following findings regarding our initial hypotheses: completing the web-based learning module significantly increased medical students’ domain-specific knowledge of spiritual care (supporting hypothesis 1) and significantly enhanced their attitudes and self-efficacy towards exploring spiritual aspects (supporting hypotheses 2a and 2b). Learning from the module significantly improved their task-related skills performance (supporting hypothesis 3). These effects are meaningful since all respective effect sizes were large.

Our findings support an increasing body of evidence implying that instructional methods presenting healthcare-related protocols with step-by-step demonstrations and aids like hints can enhance the learning of complex clinical skills [26, 30, 37–39]. This study contributes to the existing literature by demonstrating that educational tools using this scaffolding effectively engage novice learners in studying content addressing spiritual aspects in less than an hour. This is a critical insight, as instructional methods are strongly required to promote effective learning in healthcare education in general [40] and any learning domain related to pain therapy [20]. Our spiritual care learning module aligns with the growing trend of incorporating holistic care into medical education. While spiritual care is not yet a formal part of the core curriculum in Switzerland—based on the present findings—this module could be integrated into communication skills training or clinical rotations focused on chronic pain, palliative care, or patient-centered care. This integration would equip students with the skills needed to address spiritual and religious concerns in a culturally sensitive manner.

Furthermore, this study presents instruments for assessing several competencies related to exploring spiritual aspects in patients with chronic pain. For instance, our InSpiRe assessment scale for scoring student task-related skills performance proved to be implementable and demonstrated high reliability among raters with relevant training and expertise. It also correlated well with the Berlin Global rating scale, implemented as a validity measure. When healthcare professionals are encouraged to take on a task they tend to resist, it is helpful to have an instrument supporting supervisors to give exact feedback in the training situation or for students to reflect on their performance [41].

One limitation of our study is that we could not include a control group. Adding a control group, however, would have allowed us to control for even more threats to validity. A further limitation is that, although the performance test scenarios were developed by a team with expertise in chronic pain management and medical education, we acknowledge the importance of using validated vignettes in spiritual care research, as emphasized by Grabenweger et al. [42]. In future studies, we plan to incorporate expert validation and pilot testing to ensure that the cases are both diverse and methodologically sound. Another limitation is that we could not address long-term effects and thus could not provide information about optimal learning effects; the post-test was administered immediately after the completion of the module primarily for pragmatic reasons. Given the busy schedules of the participating medical students, this timing ensured full participation while allowing us to capture immediate learning outcomes. We recognize that this design focuses on short-term effects and does not account for retaining knowledge and skills over time. Future studies should investigate long-term effects and their applicability in settings with actual patients to further strengthen (or weaken) the findings. Due to the novelty of the subject, we had to develop our instruments. Although these instruments demonstrated good validity and reliability scores, they must be further tested in comparable contexts and with different raters/subjects to prove their operability and robustness against biases and other potential factors that could skew the results. This is a potential limitation, possibly affecting the replicability of the present findings. Future studies may consider using our instruments to address this issue. A subsequent point of criticism is the limitation of using the BGR scale to assess convergent validity in fully capturing competencies specific to spiritual care. The BGR scale measures general communication skills such as structure, rapport, and communication effectiveness, which overlap with aspects of spiritual care. However, its applicability for validating spiritual care instruments is somehow limited. The InSpiRe Assessment Scale developed in this study may thus require further validation in future research focused on spiritual care competencies. A final limitation is that the findings of this study are specific to medical students, which may limit the generalizability to other healthcare professionals. As novice learners, medical students have unique educational needs and limited clinical experience compared to seasoned healthcare providers. Further studies are necessary to evaluate the effectiveness of the spiritual care learning module among a broader group of professionals, such as nurses, chaplains, and experienced physicians, to assess whether the benefits observed with medical students also apply to these groups. This will help determine if the training’s impact extends across varying levels of clinical expertise and professional roles.

Our study has several strengths. First, participants were randomly selected, which supports preventing self-selection bias. Second, cognitive and behavioral outcomes were independently evaluated by blinded expert assessors, which supports ensuring objectivity. Third, performance outcomes were characterized by transferring theoretical knowledge gained through completing the web-based learning module to different simulated practice situations, which supports enhancing generalizability. Fourth, as recommended by Koenig and Carey [43], we carefully considered the conceptualization of spirituality used in this study to avoid potential contamination in measurement. Our inclusive definition of spirituality reflects its multi-dimensional nature and is consistent with current literature, allowing us to examine religious and non-religious aspects of spiritual care. This approach ensures that our findings can be compared with similar studies in the field while also contributing to a nuanced understanding of how spirituality affects coping with chronic pain.

## Conclusions

Little evidence shows how to perform the crucial clinical task of exploring spiritual aspects. With this paper, we could add that providing a web-based educational tool applying an evidence-based protocol for communication about spiritual aspects, audio-video demonstrations of its steps, and hints denoting critical actions can support initial skills learning, increase self-efficacy beliefs, and evoke positive attitude shifts towards this task, regardless of time, location, or the need of a lecturer. Moreover, we have provided promising instruments for measuring task-related competencies in this new field of investigation, as they showed good reliability and validity scores. However, they should be further tested in future studies to prove their rigor.

Summing up, the learning intervention on addressing spiritual aspects in encounters with patients with chronic pain, as examined in this study, was associated with positive effects related to cognitive, affective, and behavioral dimensions. Since the effects were practically significant, we conclude that the present findings are helpful, especially for health profession domains in which coping with chronic pain is an issue.

Despite the promising results, potential limitations exist regarding the validity of the newly developed instruments. The BGR scale used for convergent validity focuses primarily on general communication skills and may not fully capture the specific competencies related to spiritual care. Additionally, the instruments have yet to undergo extensive validation across different healthcare settings or among diverse professional groups, potentially limiting their generalizability. Future research should include validation from spiritual care experts, chaplains, and pain management specialists to ensure accuracy and test the tools’ applicability across various clinical contexts.

## Supplementary Information

Supplementary material.

## Data Availability

The datasets generated and/or analysed during the current study are available from the corresponding author on reasonable request.

## Abbreviations

InSpiRe: Integration of Spirituality and/or Religion in patient encounters
SP: Simulated patient
BGR: Berlin Global Rating scale

## Footnotes

1 In this study, the original materials, including the tests, were developed in German and subsequently translated into English for publication purposes. The translation was performed by bilingual professionals with expertise in medical education. However, due to potential nuances in translation, certain points may not have been entirely clear. For those interested, we are happy to provide the original German versions of the scales upon request to ensure both clarity and accuracy.

2 Please note that the performance test scenarios focused on general spiritual concerns related to chronic pain, rather than specific religious practices, to ensure inclusivity for the participants. However, religious aspects are an important part of the broader concept of spirituality and were covered in the theoretical component of the learning module. Future studies may include case scenarios that highlight religious coping mechanisms to further enhance the comprehensiveness of related training programs.

3 A worked example usually covers an introduction to a given problem (here: a patient suffering from pain), a task (here: addressing spiritual aspects) and a demonstration of each step required to achieve the task (here: identifying spiritual resources and distress).

4 To ensure that the item scales do match throughout the questionnaire, we have recoded given items for this analysis only.
