# Supplementary material for: Learning how to explore spiritual aspects in encounters with patients with chronic pain: a pre-test post-test trial on the effectiveness of a web-based learning intervention
Source: BMC Med Educ. 2024 Oct 25;24:1212. doi: 10.1186/s12909-024-06142-2 (PMC11515423; doi:10.1186/s12909-024-06142-2)
Supplement: Supplementary file 1 — Supplementary material. [file 12909_2024_6142_MOESM1_ESM.docx]

**Appendices**

**Appendix A. Items of the knowledge test**

*Please answer the following questions briefly by writing in the text fields. The questions are formulated in a way that shall invite you to philosophize. However, if you think you cannot add sufficiently, you may write “N/A” or “no answer” in the respective text fields.*

***Question 1:*** *Why could it be relevant to address spiritual aspects in encounters with patients with chronic pain?*

***Question 2:*** *What are the basic characteristics of spirituality and spiritual care from a healthcare-related point of view?*

***Question 3:*** *Which ways can be considered to start a patient encounter in which spiritual aspects could be addressed?*

***Question 4:*** *How can one approach encouraging patient consent in encounters where spiritual aspects could be explored?*

***Question 5:*** *In what ways can spiritual aspects in patient encounters be explored?*

***Question 6:*** *When a patient encounter including the exploration of spiritual aspects has ended: which critical questions for reflection might be helpful for the provider?*

**Appendices**

**Appendix B. Item pairs indicating the attitude towards the task of exploring spiritual aspects in patient encounters**

*We would like to learn, how you score certain attributes that might or might not play a role in the task of exploring spiritual aspects in encounters with patients with chronic pain. We are interested in your opinion; there are no wrong answers. Please score the following pairs of statements independently from one another.*

***Item pair 1:*** *Appearance*

***Statement 1a:*** *Spiritual aspects [...] emerge in encounters with patients with chronic pain. [0=never; 1=seldomly; 2=sometimes; 3=often; 4=very often].*

***Statement 1b:*** *If spiritual aspects arise during encounters with chronic pain patients, I typically find it […] to explore them. [-2 irrelevant; -1 rather irrelevant; 0=neither irrelevant nor relevant; +1=rather relevant; +2=relevant].*

***Item pair 2:*** *Positive impact*

***Statement 2a:*** *Spiritual aspects […] help patients in dealing with their chronic pain. [0=never; 1=seldomly; 2=sometimes; 3=often; 4=very often].*

***Statement 2b:*** *If spiritual aspects do help patients in dealing with their chronic pain, I find it […] to explore them in encounters. [-2 useless; -1 rather useless; 0=neither useless nor useful; +1=rather useful; +2=useful].*

***Item pair 3:*** *Negative impact*

***Statement 3a:*** *Spiritual aspects […] hinder patients in dealing with their chronic pain. [0=never; 1=seldomly; 2=sometimes; 3=often; 4=very often].*

**Appendices**

***Statement 3b:*** *If spiritual aspects do hinder patients in dealing with their chronic pain, I find it […] to explore such aspects in encounters. [-2 useless; -1 rather useless; 0=neither useless nor useful; +1=rather useful; +2=useful].*

***Item pair 4:*** *Willingness to talk*

***Statement 4a:*** *Patients with chronic pain are […] willing to talk about spiritual aspects during encounters. [0=never; 1=seldomly; 2=sometimes; 3=often; 4=very often].*

***Statement 4b:*** *If patients with chronic pain do want to talk about spiritual aspects with their doctors, I find it […] to address them in encounters. [-2 unimportant; -1 rather unimportant; 0=neither unimportant nor important; +1=rather important; +2=important].*

***Item pair 5:*** *Scientific evidence*

***Statement 5a:*** *Exploring spiritual aspects in encounters with patients with chronic pain […] is unscientific. [0=never; 1=seldomly; 2=sometimes; 3=often; 4=very often].*

***Statement 5b^[[1]](#footnote-1)^i:*** *If the exploration of spiritual aspects in encounters with patients with chronic pain is indeed unscientific, I find it […] to do so. [-2 inappropriate; -1 rather inappropriate; 0=neither inappropriate nor appropriate; +1=rather appropriate; +2= appropriate].*

**Appendices**

***Item pair 6:*** *Distraction*

***Statement 6a:*** *Exploring spiritual aspects in encounters with patients with chronic pain […] distracts from the actual treatment plan. [0=never; 1=seldomly; 2=sometimes; 3=often; 4=very often].*

***Statement 6b^[[2]](#footnote-2)^ii:*** *If the exploration of spiritual aspects in encounters with patients with chronic pain does distract from the actual treatment plan, I find it […] to do so. [-2 inappropriate; -1 rather inappropriate; 0=neither inappropriate nor appropriate; +1=rather appropriate; +2= appropriate].*

***Item pair 7:*** *Privacy*

***Statement 7a:*** *Exploring spiritual aspects in encounters with patients with chronic pain […] bothers the patients’ privacy. [0=never; 1=seldomly; 2=sometimes; 3=often; 4=very often].*

***Statement 7b:*** *If the exploration of spiritual aspects in encounters with patients with chronic pain does bother the patients’ privacy, I find it […] to do so. [-2 uncomfortable; -1 rather uncomfortable; 0=neither uncomfortable nor comfortable; +1=rather comfortable; +2= comfortable].*

**Appendices**

***Item pair 8:*** *Scope of responsibility*

***Statement 8a:*** *In the future,* *doctors will […] be tasked with exploring spiritual aspects in encounters with patients with chronic pain. [0=never; 1=seldomly; 2=sometimes; 3=often; 4=very often].*

***Statement 8b:*** *If doctors indeed will be tasked with exploring spiritual aspects in encounters with patients with chronic pain, I will […] identify myself with it. [-2 not at all; -1 rather weakly; 0=neither weakly nor strongly; +1=rather strongly; +2= strongly].*

**Appendices**

**Appendix C. Scenario A as applied for the performance test.**

**«On the move»**

**Setting:** General practitioner's practice; deputy to Dr. Müller

**Reason for consultation:** Persistent pain

**Background information:**

Mrs. Lehmann, 28 years, trauma after bicycle accident 12 years ago in the right foot. Since then, she has had several operations, the last one 1.5 years ago with 3 permanent screws (see X-ray). OSG permanently stiffened. Still suffers from pain, especially after exertion but also at rest.

**X-ray:**

**
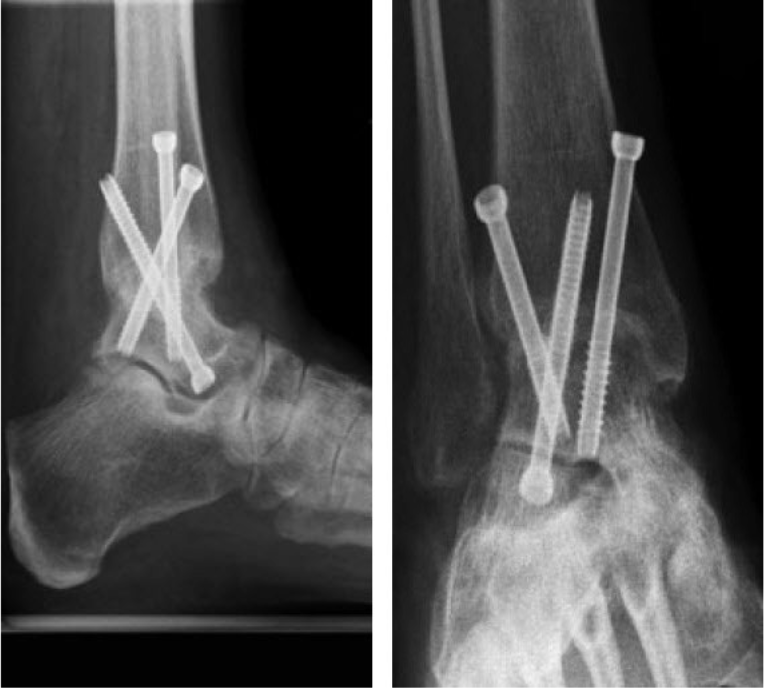
**

**Pain medication:**

Ibubrofen 600mg 1-1-1

Paracetamol 1g 1-1-1-1

Esomeprazole 20mg 1-0-0

OxyNorm 5mg up to max 6x/day

***Note:*** The painkiller dosage has already been maxed out. No morphological correlates for the pain perception could be identified, neither in the clinical examination nor the imaging.

**Your Task: SP encounter (15 minutes)**

1. Take a problem-oriented history.
2. The aim of the encounter is to identify possible resources/distress of the SP and to develop a plan for further action.

**Appendices**

**Appendix D. Scenario B as applied for the performance test.**

**«Painful experience»**

**Setting:** General practitioner's practice; deputy to Dr. Müller

**Reason for consultation:** Persistent pain

**Background information:**

Mrs. Zürcher, 28 years, polytrauma 2018 after a climbing accident, surgery of pelvic ring fracture, good healing but persistent pain in lumbar spine.

**Pain medication:**

Ibubrofen 600mg 1-1-1

Paracetamol 1g 1-1-1-1

Esomeprazole 20mg 1-0-0

OxyNorm 5mg up to max 6x/day

***Note:*** The painkiller dosage has already been maxed out. No morphological correlates for the pain perception could be identified, neither in the clinical examination nor the imaging.

**Your Task: SP encounter (15 minutes)**

1. Take a problem-oriented history.
2. The aim of the encounter is to identify possible resources/distress of the SP and to develop a plan for further action.

**Appendices**

**Appendix E. Items of the performance test: the InSpiRe Assessment Scale**

**InSpiRe Assessment Scale**

**1. Welcome**

The candidate welcomes the SP appropriately by (amongst other things) introducing him/herself, sitting down, maintaining eye contact, creating a pleasant atmosphere.

| strongly disagree | 1 | 2 | 3 | 4 | 5 | strongly agree |
| --- | --- | --- | --- | --- | --- | --- |

*Comment:*

**2. Starting the conversation**

The candidate starts the conversation appropriately by opening space for cues and concerns through asking about the SP’s status using open questions (e.g., "How have you felt over the last few days?") or (in)directly addressing it (e.g., "It seems to me that you are suffering").

| strongly disagree | 1 | 2 | 3 | 4 | 5 | strongly agree |
| --- | --- | --- | --- | --- | --- | --- |

*Comment:*

**3. Consent**

The candidate explores the SP's willingness to have a conversation about spiritual aspects by appropriately seeking for implicit or explicit approval.

| strongly disagree | 1 | 2 | 3 | 4 | 5 | strongly agree |
| --- | --- | --- | --- | --- | --- | --- |

*Comment:*

**4. Non-directed exploration**

The candidate appropriately explores spiritual aspects in a non-directed manner by e.g., asking open questions about what does help/hinder the coping process, listening attentively, mirroring, repeating what has been said in own words.

| strongly disagree | 1 | 2 | 3 | 4 | 5 | strongly agree |
| --- | --- | --- | --- | --- | --- | --- |

*Comment:*

**Appendices**

**5. Directed exploration**

The candidate appropriately identifies/acknowledges spiritual resources/distress in the conversation in a directed manner by asking specific spirituality-related questions (e.g., “Did you find ways to reproduce this feeling of being connected?”) and/or taking up what has been said (e.g., “you said you had the feeling of being punished by God. Would you tell me more about it?”).

| strongly disagree | 1 | 2 | 3 | 4 | 5 | strongly agree |
| --- | --- | --- | --- | --- | --- | --- |

*Comment:*

**6. Next steps**

The candidate appropriately discusses future steps by considering/reflecting spiritual aspects that could enhance/worsen functional coping with the pain based on the exploration findings (if applicable).

| strongly disagree | 1 | 2 | 3 | 4 | 5 | strongly agree |
| --- | --- | --- | --- | --- | --- | --- |

*Comment: Please note if the item is not applicable.*

**7. Summary**

The candidate properly summarizes the main points including the spiritual aspects that have been explored in the conversation by using understandable wording and determining whether the main points are clear to the SP.

| strongly disagree | 1 | 2 | 3 | 4 | 5 | strongly agree |
| --- | --- | --- | --- | --- | --- | --- |

*Comment:*

**8. Ending the conversation**

The candidate ends the conversation appropriately by (amongst other things) using clear and acknowledging words, thanking for the trust, ensuring availability for potential future encounters.

| strongly disagree | 1 | 2 | 3 | 4 | 5 | strongly agree |
| --- | --- | --- | --- | --- | --- | --- |

*Comment*

1. i Response values were recoded for analysis to match with the other items (the higher the score, the superior the attitude). [↑](#footnote-ref-1)
2. ii Response values were recoded for analysis to match with the other items (the higher the score, the superior the attitude). [↑](#footnote-ref-2)
